# ECM–Receptor Regulatory Network and Its Prognostic Role in Colorectal Cancer

**DOI:** 10.3389/fgene.2021.782699

**Published:** 2021-12-06

**Authors:** Stepan Nersisyan, Victor Novosad, Narek Engibaryan, Yuri Ushkaryov, Sergey Nikulin, Alexander Tonevitsky

**Affiliations:** ^1^ Faculty of Biology and Biotechnology, HSE University, Moscow, Russia; ^2^ Shemyakin-Ovchinnikov Institute of Bioorganic Chemistry, Russian Academy of Sciences, Moscow, Russia; ^3^ Medway School of Pharmacy, University of Kent, Chatham, United Kingdom; ^4^ P. Hertsen Moscow Oncology Research Institute—Branch, National Medical Research Radiological Centre, Ministry of Health of Russian Federation, Moscow, Russia; ^5^ School of Biomedicine, Far Eastern Federal University, Vladivostok, Russia; ^6^ SRC Bioclinicum, Moscow, Russia

**Keywords:** ECM–receptor, colorectal cancer, network analysis, TCGA-COAD, TCGA-READ

## Abstract

Interactions of the extracellular matrix (ECM) and cellular receptors constitute one of the crucial pathways involved in colorectal cancer progression and metastasis. With the use of bioinformatics analysis, we comprehensively evaluated the prognostic information concentrated in the genes from this pathway. First, we constructed a ECM–receptor regulatory network by integrating the transcription factor (TF) and 5’-isomiR interaction databases with mRNA/miRNA-seq data from The Cancer Genome Atlas Colon Adenocarcinoma (TCGA-COAD). Notably, one-third of interactions mediated by 5’-isomiRs was represented by noncanonical isomiRs (isomiRs, whose 5’-end sequence did not match with the canonical miRBase version). Then, exhaustive search-based feature selection was used to fit prognostic signatures composed of nodes from the network for overall survival prediction. Two reliable prognostic signatures were identified and validated on the independent The Cancer Genome Atlas Rectum Adenocarcinoma (TCGA-READ) cohort. The first signature was made up by six genes, directly involved in ECM–receptor interaction: AGRN, DAG1, FN1, ITGA5, THBS3, and TNC (concordance index 0.61, logrank test *p* = 0.0164, 3-years ROC AUC = 0.68). The second hybrid signature was composed of three regulators: hsa-miR-32-5p, NR1H2, and SNAI1 (concordance index 0.64, logrank test *p* = 0.0229, 3-years ROC AUC = 0.71). While hsa-miR-32-5p exclusively regulated ECM-related genes (COL1A2 and ITGA5), NR1H2 and SNAI1 also targeted other pathways (adhesion, cell cycle, and cell division). Concordant distributions of the respective risk scores across four stages of colorectal cancer and adjacent normal mucosa additionally confirmed reliability of the models.

## 1 Introduction

The extracellular matrix (ECM) is a noncellular component of tissue, which biochemically and structurally supports cells. The ECM is composed of different glycoproteins such as collagens, laminins, and fibronectins (Theocharis et al., 2016), and there are dozens of cellular receptors which directly interact with the components of the ECM, for example, integrins or cadherins (Barczyk et al., 2010). Interactions between the ECM and receptors on the cellular surface regulate cell behavior and play an important role in communications between cells, cell proliferation, adhesion, and migration (Nguyen-Ngoc et al., 2012; Plotnikov et al., 2012; Schlie-Wolter et al., 2013; Lange et al., 2014).

A number of studies revealed a crucial role of the ECM–receptor interaction in colorectal cancer development and metastasis formation (Stankevicius et al., 2016; Crotti et al., 2017; Maltseva and Rodin, 2018). We recently showed the contribution of *α*5 laminin in differentiation of colorectal cancer cells and chemotherapy resistance (Maltseva et al., 2020). Several works describe biomarkers and signatures for assessment of colorectal cancer prognosis based on the expression of particular genes involved in ECM–receptor interaction, including genes encoding integrins (Boudjadi et al., 2013; Gong et al., 2019), E- and P-cadherin (Sun et al., 2011; Christou et al., 2017), and different laminins (Galatenko et al., 2018). ECM-based prognostic gene signatures were constructed for gastric (Yang et al., 2020), breast (Bergamaschi et al., 2008), prostate (Pang et al., 2019), and bladder (Qing et al., 2020) cancers. However, to the best of our knowledge, no comprehensive prognostic analysis of ECM–receptor interaction–based colorectal cancer gene signatures has been done so far.

Another dimension useful for the construction of prognostic signatures is the analysis of regulatory networks (Ahmad et al., 2012; Guo et al., 2020; Nersisyan et al., 2021b). Specifically, gene expression levels could be dynamically regulated by other molecules, such as transcription factors (TFs), microRNAs (miRNAs), and others. Recently, it was shown that miRNAs are present in a cell in different variants, called miRNA isoforms (isomiRs) (Morin et al., 2008; Loher et al., 2014). As a result of imprecise enzymatic cleavage, miRNA hairpins give rise to mature forms, which differ from each other in 1–3 nucleotides at the ends of the molecule (Zhiyanov et al., 2021). Importantly, the targetome of isomiRs with differences at 5’-ends (5’-isomiRs) significantly differ from the canonical form (Tan et al., 2014; van der Kwast et al., 2020). Thus, 5’-isomiRs could be considered separate miRNAs with their own sets of targets.

In this work, we analyzed expression patterns of genes involved in the ECM–receptor interaction pathway using RNA sequencing data of colorectal cancer samples taken from The Cancer Genome Atlas Colon Adenocarcinoma (TCGA-COAD) and Rectum Adenocarcinoma (TCGA-READ) projects (Network, 2012). First, we constructed and analyzed a regulatory network to infer 5’-isomiRs and TFs, which are direct regulators of genes from the ECM–receptor interaction pathway. The network was built with miRGTF-net—the recently developed tool which integrates both expression and database-level data for the network construction (Nersisyan et al., 2021b). Next, the obtained network was used to construct hybrid isomiR-gene signatures for predicting overall survival in colorectal cancer. For this analysis, we employed a novel technique of exhaustive search-based Cox model fitting. Namely, ExhauFS software (Nersisyan et al., 2021c) was used to construct prognostic models for all gene/5’-isomiR pairs, triples, etc; then, the best performing model was picked.

## 2 Methods

### 2.1 TCGA mRNA and miRNA Sequencing Data

RNA and miRNA sequencing read count tables were downloaded from the GDC Data Portal for *n* = 426 TCGA-COAD and *n* = 161 TCGA-READ colorectal cancer samples (tumors with unmatched miRNA/mRNA profiles or without clinical information were not considered). For the comparison of primary tumors and adjacent normal mucosa, *n* = 7 TCGA-COAD normal samples were also included. With the use of the trimmed mean of M-values (TMM) algorithm implemented in the edgeR v3.30.3 package (Robinson et al., 2010), the obtained mRNA-seq and miRNA-seq count matrices were processed into the TMM-FPKM and TMM-RPM tables, respectively. Low expressed genes and miRNAs were filtered out using the default procedure available in edgeR.

The conventional nomenclature was used to annotate 5’-isomiRs (Telonis et al., 2015; Zhiyanov et al., 2021). For example, hsa-miR-30e-5p|+1 stands for the mature hsa-miR-30e-5p miRNA without the first nucleotide at the 5’-end (i.e., the number after | represents the offset at the 5’-end in the direction from the 5’-end to the 3’-end).

### 2.2 Network Analysis

A recently developed miRGTF-net tool (Nersisyan et al., 2021b) was applied to the TCGA-COAD dataset for the construction of a colorectal cancer miRNA-gene-TF regulatory network. The main feature of this approach consists in the integration of expression data (TCGA-COAD) with the biological interaction databases:

• TFLink database (https://tflink.net) was used to extract TF-gene interactions;
• TF-miRNA interactions were obtained from TransmiR v2.0 (Tong et al., 2019);
• miRDB v6.0 (Chen and Wang, 2020) custom prediction mode was employed to predict targets of 5′-isomiRs (as recommended by the tool authors, interactions with target scores ≥80 were considered).

First, the initial network was constructed based on the interactions listed in these databases. Second, all uncorrelated and wrong-directional edges (like positively correlated miRNAs and their targets) were discarded. Then, interaction scores were assigned to each edge and node of the network. The interaction scores are based on the strength of the linear dependence between expressions levels of the connected nodes. After filtering out nodes and edges with low interaction scores, the resulting network consisted of nodes with significant influence on some other nodes and/or significantly regulated by some other node. Configuration for miRGTF-net execution is listed in Supplementary Material.

ECM–receptor interaction–related genes were taken from the KEGG hsa04512 pathway (Kanehisa et al., 2021) (we refer to these genes as *ECM set*). Next, the output of miRGTF-net was used to construct a subnetwork composed of molecules, which either regulates a gene from the ECM–receptor interaction pathway or is regulated by a gene from the pathway (*ECM+ set*).

### 2.3 Construction of Prognostic Signatures

The TCGA-COAD cohort was split into training (75% of samples) and filtration (25%) sets with a stratification by outcome indicator (death or censoring) and overall survival time (date of death or date of the last follow-up). Namely, we first sorted samples by outcome indicator and then sorted samples by overall survival time within each outcome group. Finally, every fourth sample was added to the filtration set, and the resting samples were labeled as the training ones. The independent TCGA-READ dataset was used for the model validation (test set).

The ExhauFS tool (Nersisyan et al., 2021c) was used to fit Cox survival regression models. For each length of prognostic signature (*k* = 1, 2, … , 10), we first selected *n* most individually predictive features (see the next paragraph for the details) and then fit Cox models for all possible $\left(\begin{array}{c} n\\ k \end{array}\right)$ feature subsets. The values of *n* were chosen for reasons of limiting the computational time by the default procedure available in ExhauFS (Supplementary Table S1). The pipeline was executed in two modes: in the first one, genes from the ECM set were pre-selected, and in the second run, all 537 genes and isomiRs from the ECM+ set were considered for the model construction.

The concordance index was used as the main model accuracy metric, including the feature selection step. That is, features (genes and isomiRs) were selected according to the concordance index of the respective univariate model. In addition, patients were separated into high- and low-risk groups (the median risk score calculated on the training set was used as a cut-off value). This allowed us to construct Kaplan–Meier curves and compare low- and high-risk groups with the hazard ratio metric and the logrank test. Finally, time-dependent ROC AUC was calculated to measure discriminative power of models for predicting 3-year patient survival. Configuration for ExhauFS execution is listed in Supplementary Material.

The set of reliable models was defined by the following thresholds, set on both training and filtration sets: concordance index >0.6 , hazard ratio >2 , logrank test *p*-value <0.01 , and 3-year ROC AUC >0.6 . The best performing model was chosen by taking the signature with the maximal concordance index on the training set.

### 2.4 Enrichment Analysis

Enrichment analysis of gene sets was conducted using DAVID v6.8 (Huang et al., 2009). Significantly enriched terms were identified by setting a 0.05 threshold on false discovery rates (FDRs).

### 2.5 Statistical Analysis

A hypergeometric test was applied to

• identify regulators (TFs and isomiRs) with an overrepresented number of target genes in the ECM set;
• identify genes and isomiRs, which were overrepresented in the reliable prognostic signatures.

“Over”-regulated genes from the ECM set were determined by the binomial test. In all the cases, the Benjamini–Hochberg procedure was employed to adjust for multiple testing correction. SciPy implementation of statistical tests was used (Virtanen et al., 2020).

## 3 Results

### 3.1 Regulatory Network of ECM–Receptor Interaction Pathway

The first step of our analysis was the inference of regulatory interactions affecting genes from the ECM–receptor interaction pathway (from here onward, we refer to these genes as *ECM set*). The MiRGTF-net tool allows one to construct miRNA–gene–TF interaction networks combining both database-level and integrative miRNA/gene expression data. At the beginning, the database-level network was constructed; it contained interactions of the three types:

• TFs regulating target genes;
• TFs regulating target miRNAs;
• 5’-isomiRs downregulating target genes.

Then, TCGA-COAD gene and isomiR expression data were analyzed to filter only those interactions which are supported by a significant correlation in considered samples.

**FIGURE 1:**
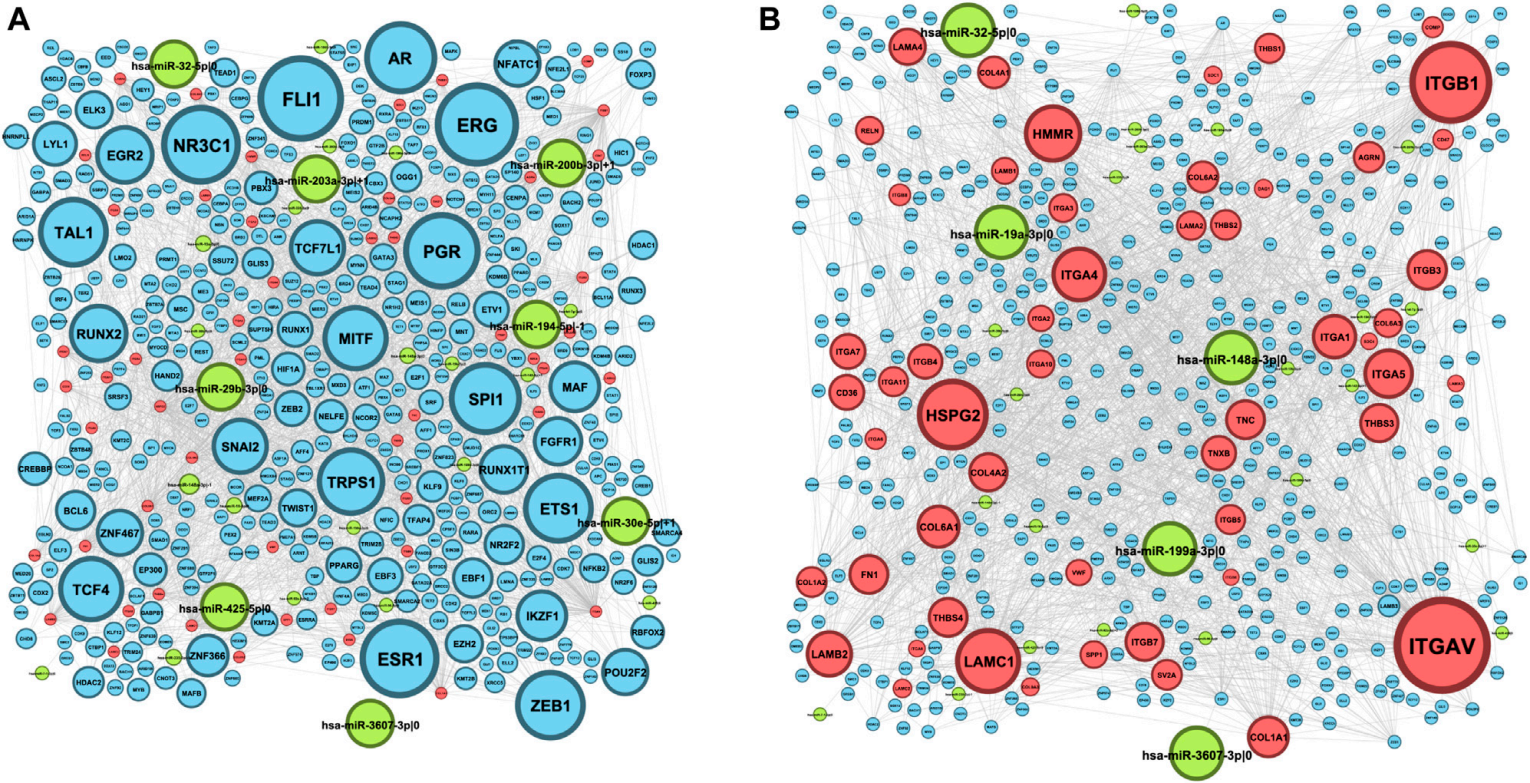
ECM–receptor regulatory network. Blue nodes represent TFs, red nodes represent genes, and green nodes represent 5′-isomiRs. The node size indicates the value of the node degree; **(A)** out-degree; **(B)** in-degree.

The resulting ECM–receptor regulatory network consisted of 522 nodes, which included 442 TFs, 27 5’-isomiRs, and 53 genes from the ECM–receptor interaction pathway (here onward, *ECM+ set*). First, we analyzed out-degrees of the network nodes, that is, the numbers of regulatory interactions outgoing from TFs and isomiRs. The network had 49 hubs—regulators whose targets were significantly enriched by the ECM set (Figure 1A, Supplementary Table S2). Aside from well-known TFs, regulating hundreds and thousands of genes (e.g., ZEB1, TWIST1, SPI1, etc.), there were three isomiRs (hsa-miR-148a-3p|-1, hsa-miR-29b-3p|0, and hsa-miR-32–5p|0) narrowly regulating the ECM set. Reciprocally, network in-degree analysis revealed nine “over”-regulated genes from the ECM set, mainly integrins and laminins (Figure 1B, Supplementary Table S3).

Interestingly, one-third of isomiR–target gene interactions (13 out of 39) were mediated by noncanonical 5’-isomiRs (i.e., isomiRs whose 5’-ends do not match with canonical the miRBase version). This included four mRNA targets of hsa-miR-148a-3p|-1 (LAMA4, LAMB2, ITGA11, and COL4A1), two targets of hsa-miR-335-3p|-1 and hsa-miR-30e-5p|+1 (both isomiRs regulated ITGA1 and COL1A2), and five isolated isomiR–gene interactions: hsa-miR-92a-3p|+2 and ITGAV, hsa-miR-203a-3p|+1 and COL4A1, hsa-miR-200b-3p|+1 and LAMA4, hsa-miR-194-5p|-1 and ITGA2, and hsa-miR-142-3p|+1 and LAMC1. Thus, consideration of 5’-isomiRs as distinct functional units added much information about RNA interference–mediated gene silencing.

### 3.2 Prognostic Power of the ECM and ECM+ Sets

Next, we assessed whether it is possible to find an accurate overall survival prediction model constructed of molecules from the ECM and ECM+ sets. We used our recently developed ExhauFS tool to go over all possible prognostic signatures composed of ECM/ECM+ genes and 5’-isomiRs, where the signature length varied from 1 to 10. While it was possible to search over all possible gene/isomiR pairs composed of 537 molecules (cardinality of the ECM+ set), the exhaustive search was computationally infeasible already for the triples. To tackle this problem, ExhauFS selects the relevant number of the most individually informative features and then performs exhaustive search among them (see Methods for the details).

**FIGURE 2:**
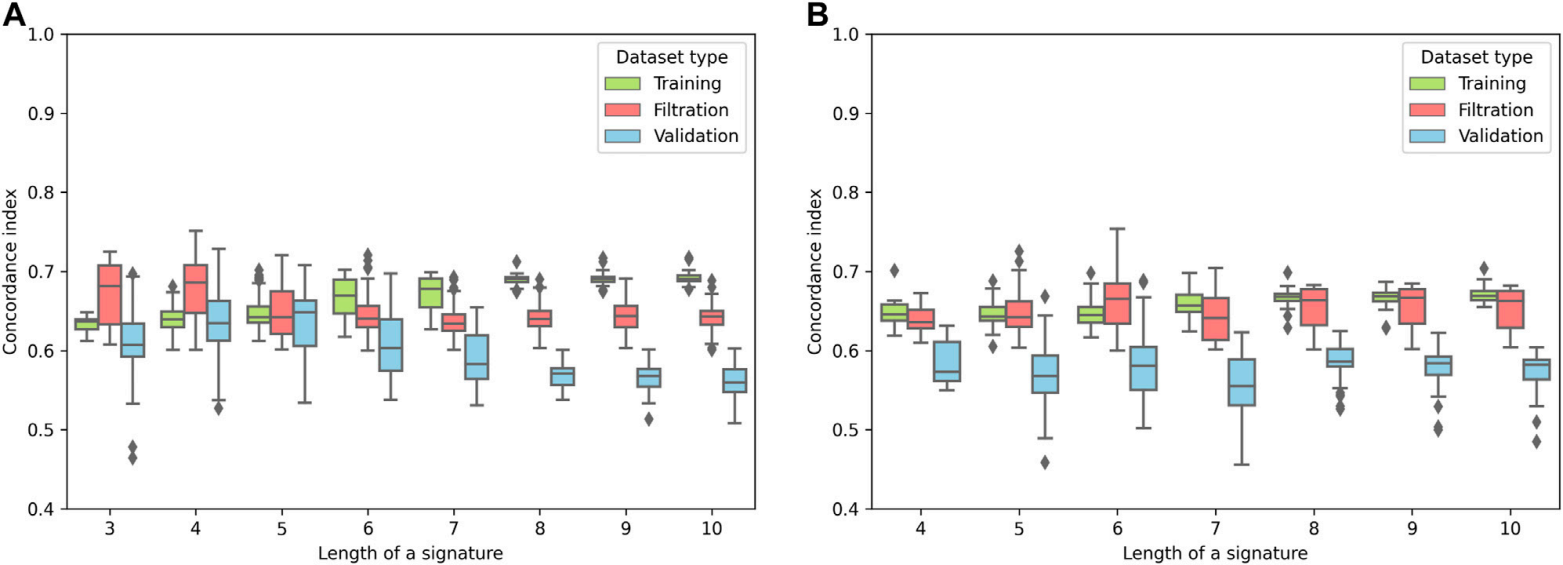
Distribution of the concordance indices of the reliable models for different lengths of signatures. **(A)** ECM+ models. **(B)** ECM models.

For the pipeline evaluation, 75% of the TCGA-COAD cohort was used for the Cox model training, and the remaining 25% was used for the filtration. The TCGA-READ dataset was used as an independent validation set. We set up several accuracy thresholds to discard models which demonstrated unreliable quality either on the training or the filtration sets (see Methods). The distribution of model accuracies (concordance indices) for each signature length (*k*) is shown in Figure 2. As it can be seen, both ECM+ and ECM models started to overfit from some point: quality of the models monotonically increased on the training set and started to drop on the filtration set after a certain signature length. The filtration set accuracy peak for the ECM+ set fell on gene/isomiR triples and quadruples (Figure 2); for the downstream analysis, we selected the shorter signatures, since there was no statistically significant difference between concordance indices for *k* = 3 and *k* = 4 (Mann–Whitney *U*-test *p* = 0.11). As for the ECM set, the highest filtration concordance indices were detected for the 6-gene signatures.

Among the ECM+ triples, the best model (according to the concordance index on the training set) was constructed with one canonical miRNA and two TFs: hsa-miR-32-5p|0, NR1H2, and SNAI1. The risk score (RS) for the model was calculated as follows:

RS=0.25∗hsa‐miR‐32‐5p|0+0.34∗NR1H2+0.25∗SNAI1.

**FIGURE 3:**
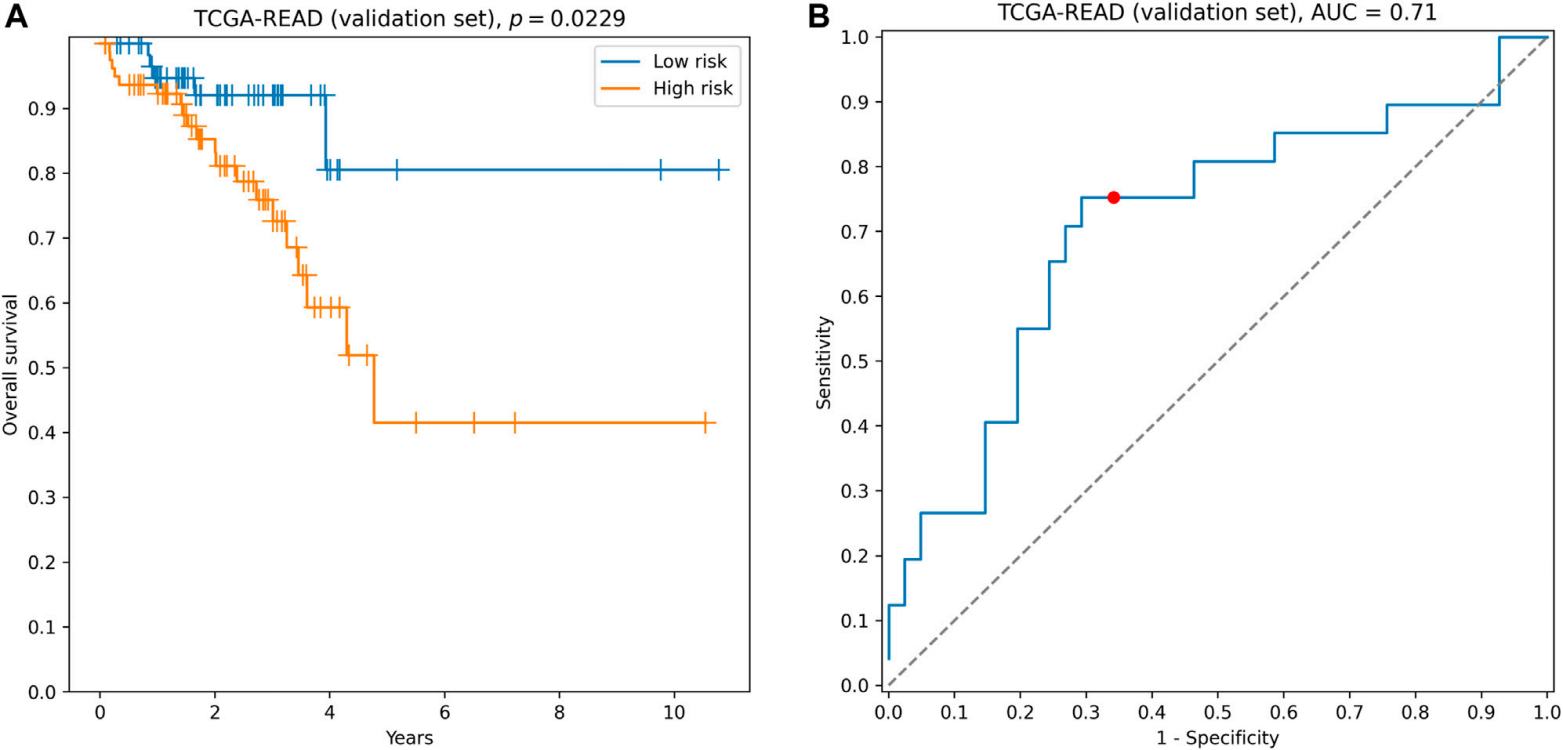
Performance of hsa-miR-32-5p**|**0, NR1H2, and SNAI1 signature on the validation set. **(A)** Kaplan–Meier curves. **(B)** 3-year ROC curve. Red point on the ROC curve corresponds to the risk score threshold, calculated as a median score on the training set.

The signature demonstrated reliable performance on the TCGA-READ validation set: the concordance index was equal to 0.64, difference in survival between groups of low- and high-risk was statistically significant (hazard ratio = 2.25, logrank test *p* = 0.0229, Figure 3A), and the model accurately classified 3-year patient survival (3-year ROC AUC = 0.71, Figure 3B).

Similarly to the ECM+ case, the most reliable signature in the ECM set was identified as follows:

RS=0.30∗AGRN−0.57∗DAG1+0.11∗FN1+0.36∗ITGA5+0.29∗THBS3−0.42∗TNC.

**FIGURE 4:**
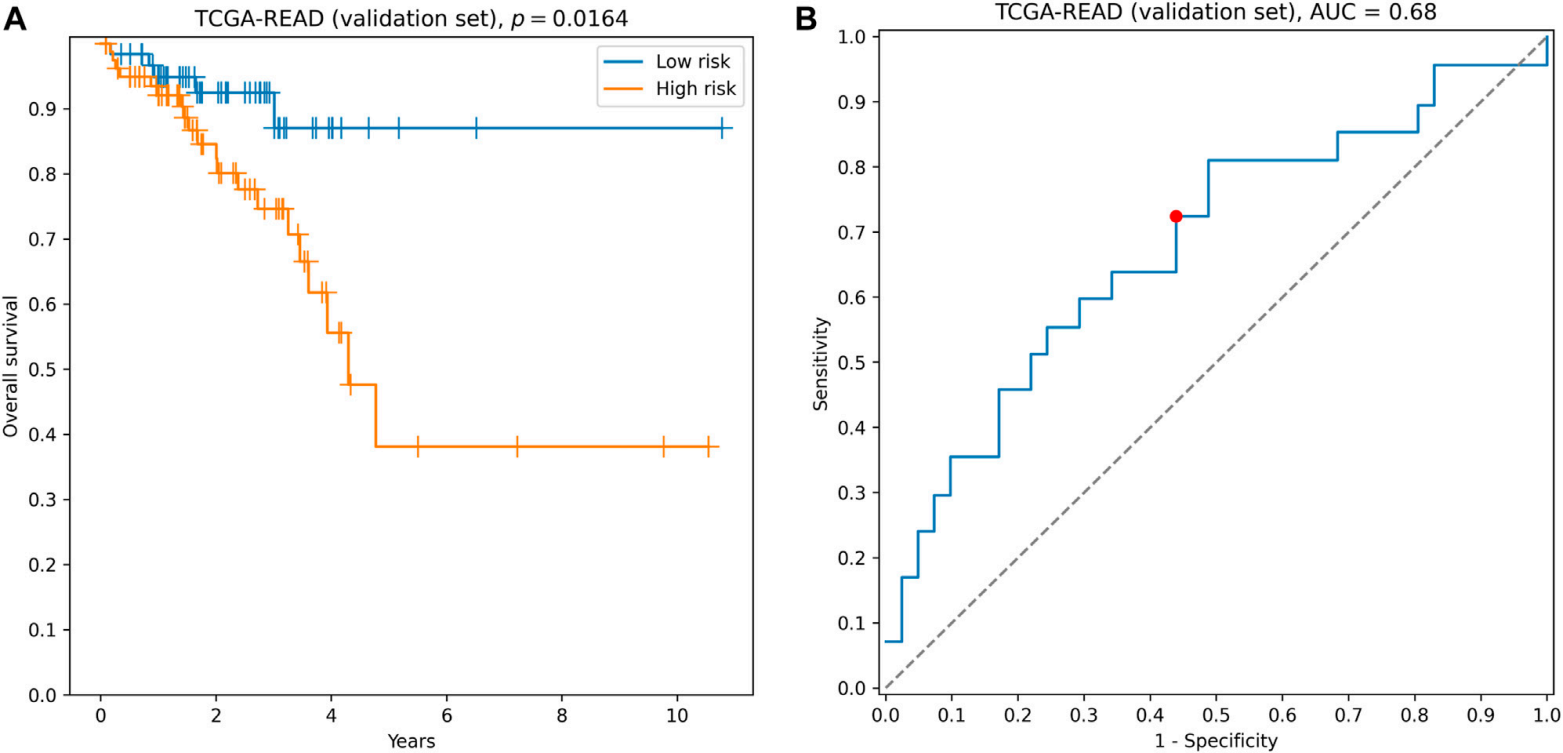
Performance of AGRN, DAG1, FN1, ITGA5, THBS3, and TNC signature on the validation set. **(A)** Kaplan–Meier curves. **(B)** 3-year ROC curve. Red point on the ROC curve corresponds to the risk score threshold, calculated as a median score on the training set.

The quality of this signature, composed of six genes directly involved in ECM–receptor interaction, was comparable to the quality of hybrid ECM+ prognostic triple: concordance index = 0.61, hazard ratio = 2.14, logrank test *p* = 0.0164 (Figure 4A), 3-year ROC AUC = 0.68 (Figure 4B). The complete list of accuracy metrics (including training and filtration sets) is presented in Supplementary Table S4 and Supplementary Figure S1.

**FIGURE 5:**
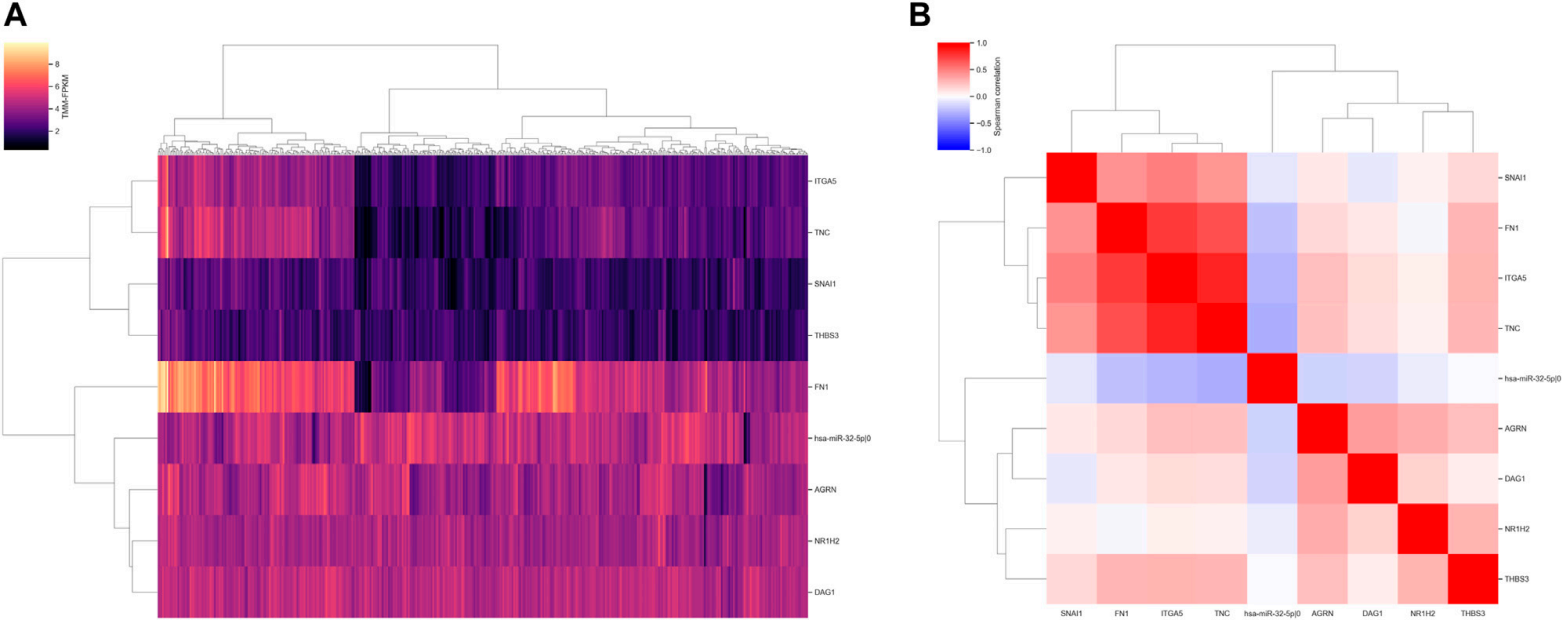
Expression distribution of the nine prognostic molecules. **(A)** Normalized and log _2_-transformed expression units. **(B)** Spearman correlation matrix.

**FIGURE 6:**
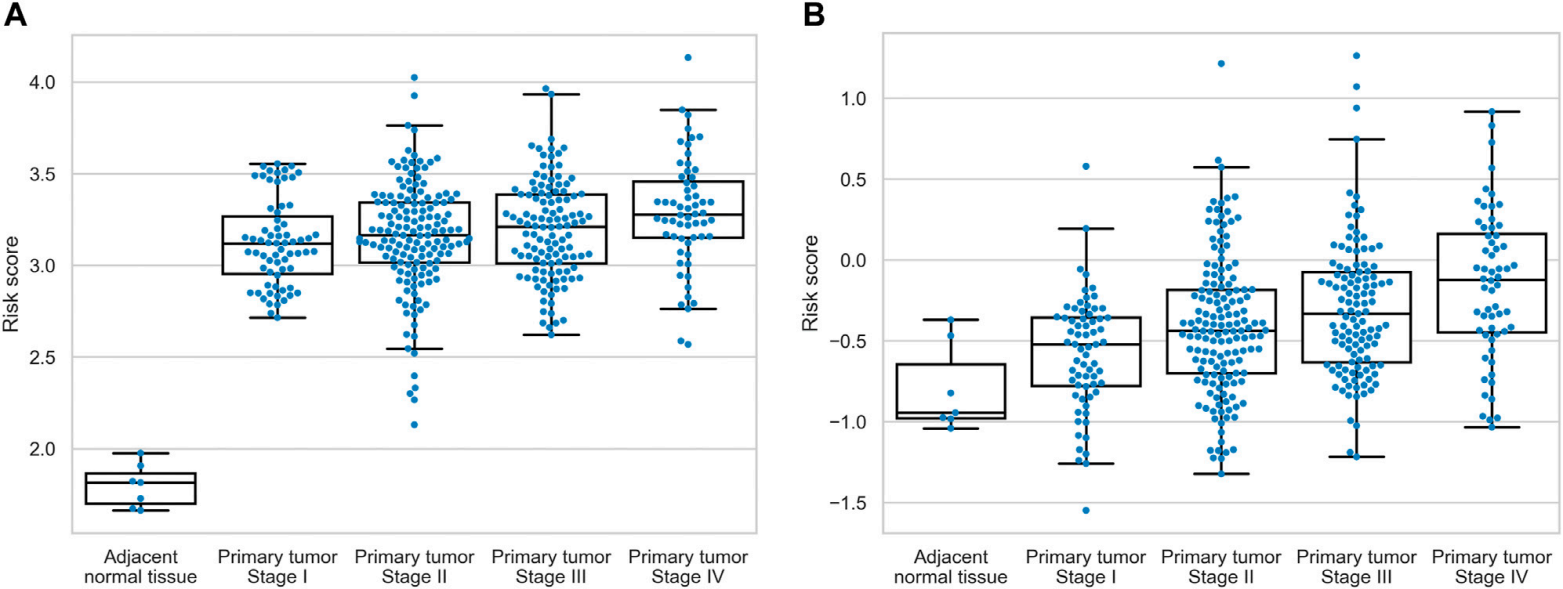
Distribution of the risk scores across normal tissues and four stages of colorectal cancer. **(A)** hsa-miR-32-5p|0, NR1H2, and SNAI1 signature. **(B)** AGRN, DAG1, FN1, ITGA5, THBS3, and TNC signature.

To assess the relationship between expression levels of nine identified prognostic molecules, we performed hierarchical clustering using both sample-wise expression values (Figure 5A) and correlation matrix (Figure 5B). Notably, three genes (FN1, ITGA5, and TNC) showed a strong co-expression pattern, while the other molecules did not form clear cluster structures. For both signatures, we compared the distributions of the underlying risk scores between four stages of colorectal cancer and adjacent normal tissues. In all cases, the risk scores monotonically increased from the normal mucosa to stage IV cancer (Figure 6). This observation is an additional piece of evidence of reliability of two constructed models.

### 3.3 Regulatory Neighborhood of the Prognostic 5’-isomiR/Gene Triple

**FIGURE 7:**
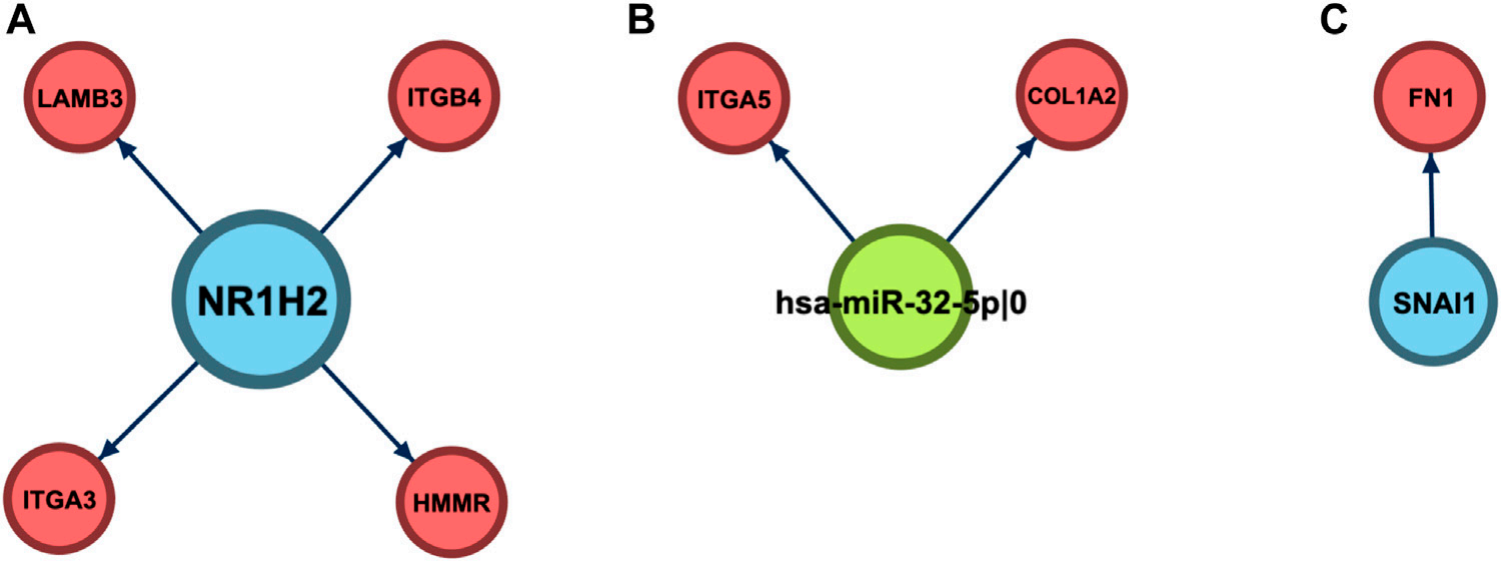
Regulatory neighborhood of the prognostic 5′-isomiR/gene triple. **(A)** NR1H2. **(B)** hsa-miR-32–5p|0. **(C)** SNAI1.

Since the best ECM+ model was composed of three regulators (one miRNA and two TFs), the next step of our analysis was to explore the landscape of regulatory interactions mediated by these molecules (Figure 7). Out of three regulators, only miR-32 specifically regulated the ECM–interaction pathway: two (COL1A2 and ITGA5) out of nine predicted targets were from the ECM set (adjusted *p* = 8.92 × 10^–3^). Moreover, DAVID enrichment analysis of this set of nine genes revealed only two pathways tightly related to the ECM: ECM–receptor interaction (KEGG hsa04512, adjusted *p* = 0.0168) and focal adhesion (KEGG hsa04510, *p* = 0.0463).

Unlike miR-32, targetomes of NR1H2 and SNAI1 were not focused on the ECM set; only 4/705 targets of NR1H2 (HMMR, ITGA3, ITGB4, and LAMB3, *p* = 0.86) and 1/6 targets of SNAI1 (FN1, *p* = 0.16) were associated with ECM–receptor interaction. To uncover the regulatory role of NR1H2 and SNAI1 in colorectal cancer, we also performed DAVID functional enrichment analysis of the sets of their target genes inferred by miRGTF-net. Multiple pathways were enriched in the set of NR1H2 targets, including cell cycle, cell division, DNA repair, and RNA splicing (SupplementaryTable S5). In case of SNAI1, the cell adhesion pathway was enriched when no multiple testing correction was applied (Supplementary Table S5). Thus, the inclusion of regulators in the prognostic signatures expanded the scope of the considered ECM pathway.

## 4 Discussion

In this work, we used expression data of genes constituting the ECM–receptor interaction pathway and its direct 5’-isomiR and TF regulators to compose prognostic signatures for colorectal cancer. The novel feature of the network construction step consisted in accounting for 5’-isomiR targeting. Importantly, one-third of all isomiR–gene interactions were mediated by noncanonical 5’-isomiRs. These numbers are in agreement with previous experimental findings, which demonstrated biological activity of noncanonical 5’-isomiRs, for example, miR-411|-1 (van der Kwast et al., 2020) or miR-9|+1 (Tan et al., 2014).

With the use of the ECM–receptor regulatory network, we constructed two reliable signatures for overall survival prediction. The first hybrid signature was composed of one canonical miRNA and two TFs: hsa-miR-32-5p|0, NR1H2, and SNAI1. The second signature was composed of six genes, directly involved in ECM–receptor interaction: AGRN, DAG1, FN1, ITGA5, THBS3, and TNC. A number of studies already highlighted the role of several markers from the constructed signatures in colorectal cancer. In two recent reports, miR-32 was shown to promote tumorigenesis, radioresistance, migration, and invasion of colorectal cancer by targeting BMP5 and TOB1 (Chen et al., 2018; Liang et al., 2019). With the use of sequence-based target prediction coupled with co-expression analysis, here, we first showed that miR-32 targets are overrepresented in the ECM–receptor interaction pathway (adjusted *p*-value = 8.92 × 10^–3^). Thus, the new possible regulatory role of miR-32 was uncovered. Another member of the ECM+ prognostic triple, the SNAI1 transcription factor, was also linked to the poor prognosis of colorectal cancer. Namely, SNAI1 regulates epithelial–mesenchymal transition (EMT) by suppressing E-cadherin and promotes chemoresistance in colorectal cancer (Hoshino et al., 2009; Wang et al., 2018). Nevertheless, we did not find specific roles of NR1H2 in the colorectal cancer development, progression, or metastasis. Functional enrichment analysis of NR1H2 downstream targets suggested the contribution of this TF to the regulation of core cellular pathways, such as cell cycle and cell division.

The second signature was also partially composed of well-studied genes. We previously showed the strong upregulation of ITGA5 in Caco-2 human colorectal cancer cell lines exposed to hypoxia, as a consequence of hypoxia-induced decrease in expression of its direct regulator—miR-148a (Nersisyan et al., 2021a). Notably, this regulatory interaction (miR-148a suppressing ITGA5) was also supported by the negative correlation in our miRGTF-net analysis. In the same work, the negative association between ITGA5 expression levels and patients’ overall survival was observed. Other studies showed that reduced DAG1 protein expression is associated with poor outcome of colorectal cancer (Coco et al., 2012); downregulation of FN1 decreases proliferation, migration, and invasion of colorectal cancer cells (Cai et al., 2018), while TNC induces EMT and proliferation (Yang et al., 2018). As for AGRN and THBS3, we have not found evidence on their role in colorectal cancer pathogenesis. The comprehensive reference list summarizing the role of the selected genes in colorectal cancer prognosis is presented in Supplementary Table S6.

## Data Availability

The original contributions presented in the study are included in the article/Supplementary Material; further inquiries can be directed to the corresponding author.

## References

[B1] Ahmad F. K. Deris S. Othman N. H. (2012). The Inference of Breast Cancer Metastasis through Gene Regulatory Networks. J. Biomed. Inform. 45, 350–362. 10.1016/j.jbi.2011.11.015 22179053

[B2] Barczyk M. Carracedo S. Gullberg D. (2010). Integrins. Cell Tissue Res 339, 269–280. 10.1007/s00441-009-0834-6 19693543 PMC2784866

[B3] Bergamaschi A. Tagliabue E. Sørlie T. Naume B. Triulzi T. Orlandi R. (2008). Extracellular Matrix Signature Identifies Breast Cancer Subgroups with Different Clinical Outcome. J. Pathol. 214, 357–367. 10.1002/path.2278 18044827

[B4] Boudjadi S. Carrier J. C. Beaulieu J.-F. (2013). Integrin α1 Subunit Is Up-Regulated in Colorectal Cancer. Biomark Res. 1, 16. 10.1186/2050-7771-1-16 24252313 PMC4177608

[B5] Cai X. Liu C. Zhang T. N. Zhu Y. W. Dong X. Xue P. (2018). Down‐regulation of FN1 Inhibits Colorectal Carcinogenesis by Suppressing Proliferation, Migration, and Invasion. J. Cel. Biochem. 119, 4717–4728. 10.1002/jcb.26651 29274284

[B6] Chen E. Li Q. Wang H. Zhang P. Zhao X. Yang F. (2018). MiR-32 Promotes Tumorigenesis of Colorectal Cancer by Targeting BMP5. Biomed. Pharmacother. 106, 1046–1051. 10.1016/j.biopha.2018.07.050 30119170

[B7] Chen Y. Wang X. (2020). miRDB: an Online Database for Prediction of Functional microRNA Targets. Nucleic Acids Res. 48, D127–D131. 10.1093/nar/gkz757 31504780 PMC6943051

[B8] Christou N. Perraud A. Blondy S. Jauberteau M.-O. Battu S. Mathonnet M. (2017). E-cadherin: A Potential Biomarker of Colorectal Cancer Prognosis. Oncol. Lett. 13, 4571–4576. 10.3892/ol.2017.6063 28588719 PMC5452924

[B9] Coco C. Zannoni G. F. Caredda E. Sioletic S. Boninsegna A. Migaldi M. (2012). Increased Expression of CD133 and Reduced Dystroglycan Expression Are strong Predictors of Poor Outcome in colon Cancer Patients. J. Exp. Clin. Cancer Res. 31, 71. 10.1186/1756-9966-31-71 22964035 PMC3541988

[B10] Crotti S. Piccoli M. Rizzolio F. Giordano A. Nitti D. Agostini M. (2017), Extracellular Matrix and Colorectal Cancer: How Surrounding Microenvironment Affects Cancer Cell Behavior? J. Cel. Physiol. 232, 967–975. 10.1002/jcp.25658 27775168

[B11] Galatenko V. V. Maltseva D. V. Galatenko A. V. Rodin S. Tonevitsky A. G. (2018). Cumulative Prognostic Power of Laminin Genes in Colorectal Cancer. BMC Med. Genomics 11, 9. 10.1186/s12920-018-0332-3 29504916 PMC5836818

[B12] Gong Y. Ruan G. Liao X. Wang X. Liao C. Wang S. (2019). Diagnostic and Prognostic Values of Integrin *α* Subfamily mRNA Expression in colon Adenocarcinoma. Oncol. Rep. 10.3892/or.2019.7216 PMC6667841 31322253

[B13] Guo Q. Wang J. Gao Y. Li X. Hao Y. Ning S. (2020). Dynamic TF-lncRNA Regulatory Networks Revealed Prognostic Signatures in the Development of Ovarian Cancer. Front. Bioeng. Biotechnol. 8. 10.3389/fbioe.2020.00460 PMC7237576 32478062

[B14] Hoshino H. Miyoshi N. Nagai K.-i. Tomimaru Y. Nagano H. Sekimoto M. (2009). Epithelial-mesenchymal Transition with Expression of SNAI1-Induced Chemoresistance in Colorectal Cancer. Biochem. Biophysical Res. Commun. 390, 1061–1065. 10.1016/j.bbrc.2009.10.117 19861116

[B15] Huang D. W. Sherman B. T. Lempicki R. A. (2009). Systematic and Integrative Analysis of Large Gene Lists Using DAVID Bioinformatics Resources. Nat. Protoc. 4, 44–57. 10.1038/nprot.2008.211 19131956

[B16] Kanehisa M. Furumichi M. Sato Y. Ishiguro-Watanabe M. Tanabe M. (2021). KEGG: Integrating Viruses and Cellular Organisms. Nucleic Acids Res. 49, D545–D551. 10.1093/nar/gkaa970 33125081 PMC7779016

[B17] Lange T. Samatov T. R. Tonevitsky A. G. Schumacher U. (2014). Importance of Altered Glycoprotein-Bound N- and O-Glycans for Epithelial-To-Mesenchymal Transition and Adhesion of Cancer Cells. Carbohydr. Res. 389, 39–45. 10.1016/j.carres.2014.01.010 24491280

[B18] Liang H. Tang Y. Zhang H. Zhang C. (2019). MiR-32-5p Regulates Radiosensitization, Migration and Invasion of Colorectal Cancer Cells by Targeting TOB1 Gene. Ott Vol. 12, 9651–9661. 10.2147/OTT.S228995 PMC6861524 31814731

[B19] Loher P. Londin E. R. Rigoutsos I. (2014). IsomiR Expression Profiles in Human Lymphoblastoid Cell Lines Exhibit Population and Gender Dependencies. Oncotarget 5, 8790–8802. 10.18632/oncotarget.2405 25229428 PMC4226722

[B20] Maltseva D. Raygorodskaya M. Knyazev E. Zgoda V. Tikhonova O. Zaidi S. (2020). Knockdown of the α5 Laminin Chain Affects Differentiation of Colorectal Cancer Cells and Their Sensitivity to Chemotherapy. Biochimie 174, 107–116. 10.1016/j.biochi.2020.04.016 32334043

[B21] Maltseva D. V. Rodin S. A. (2018). Laminins in Metastatic Cancer. Mol. Biol. 52, 350–371. 10.1134/s0026893318030093 29989574

[B22] Morin R. D. O'Connor M. D. Griffith M. Kuchenbauer F. Delaney A. Prabhu A.-L. (2008). Application of Massively Parallel Sequencing to microRNA Profiling and Discovery in Human Embryonic Stem Cells. Genome Res. 18, 610–621. 10.1101/gr.7179508 18285502 PMC2279248

[B23] Nersisyan S. Galatenko A. Chekova M. Tonevitsky A. (2021a). Hypoxia-Induced miR-148a Downregulation Contributes to Poor Survival in Colorectal Cancer. Front. Genet. 12. 10.3389/fgene.2021.662468 PMC8202010 34135940

[B24] Nersisyan S. Galatenko A. Galatenko V. Shkurnikov M. Tonevitsky A. (2021b). miRGTF-Net: Integrative miRNA-Gene-TF Network Analysis Reveals Key Drivers of Breast Cancer Recurrence. PLOS ONE 16, e0249424. 10.1371/journal.pone.0249424 33852600 PMC8046230

[B25] Nersisyan S. Novosad V. Galatenko A. Sokolov A. Bokov G. Konovalov A. (2021c). Exhaufs: Exhaustive Search-Based Feature Selection for Classification and Survival Regression. bioRxiv. 10.1101/2021.08.03.454798 PMC8976470 35378930

[B26] Network T. C. G. A. (2012). Comprehensive Molecular Characterization of Human colon and Rectal Cancer. Nature 487, 330–337. 10.1038/nature11252 22810696 PMC3401966

[B27] Nguyen-Ngoc K.-V. Cheung K. J. Brenot A. Shamir E. R. Gray R. S. Hines W. C. (2012). ECM Microenvironment Regulates Collective Migration and Local Dissemination in normal and Malignant Mammary Epithelium. Proc. Natl. Acad. Sci. 109, E2595–E2604. 10.1073/pnas.1212834109 22923691 PMC3465416

[B28] Pang X. Xie R. Zhang Z. Liu Q. Wu S. Cui Y. (2019). Identification of SPP1 as an Extracellular Matrix Signature for Metastatic Castration-Resistant Prostate Cancer. Front. Oncol. 9, 924. 10.3389/fonc.2019.00924 31620371 PMC6760472

[B29] Plotnikov S. V. Pasapera A. M. Sabass B. Waterman C. M. (2012). Force Fluctuations within Focal Adhesions Mediate ECM-Rigidity Sensing to Guide Directed Cell Migration. Cell 151, 1513–1527. 10.1016/j.cell.2012.11.034 23260139 PMC3821979

[B30] Qing L. Gu P. Liu M. Shen J. Liu X. Guang R. (2020). Extracellular Matrix-Related Six-lncRNA Signature as a Novel Prognostic Biomarker for Bladder Cancer. Ott Vol. 13, 12521–12538. 10.2147/ott.S284167 PMC7733340 33324071

[B31] Robinson M. D. McCarthy D. J. Smyth G. K. (2010). edgeR: a Bioconductor Package for Differential Expression Analysis of Digital Gene Expression Data. Bioinformatics 26, 139–140. 10.1093/bioinformatics/btp616 19910308 PMC2796818

[B32] Schlie-Wolter S. Ngezahayo A. Chichkov B. N. (2013). The Selective Role of ECM Components on Cell Adhesion, Morphology, Proliferation and Communication *In Vitro* . Exp. Cel Res. 319, 1553–1561. 10.1016/j.yexcr.2013.03.016 23588204

[B33] Stankevicius V. Vasauskas G. Noreikiene R. Kuodyte K. Valius M. Suziedelis K. (2016). Extracellular Matrix-dependent Pathways in Colorectal Cancer Cell Lines Reveal Potential Targets for Anticancer Therapies. Ar 36, 4559–4568. 10.21873/anticanres.11004 27630296

[B34] Sun L. Hu H. Peng L. Zhou Z. Zhao X. Pan J. (2011). P-cadherin Promotes Liver Metastasis and Is Associated with Poor Prognosis in Colon Cancer. Am. J. Pathol. 179, 380–390. 10.1016/j.ajpath.2011.03.046 21703417 PMC3123784

[B35] Tan G. C. Chan E. Molnar A. Sarkar R. Alexieva D. Isa I. M. (2014). 5′ isomiR Variation Is of Functional and Evolutionary Importance. Nucleic Acids Res. 42, 9424–9435. 10.1093/nar/gku656 25056318 PMC4132760

[B36] Telonis A. G. Loher P. Jing Y. Londin E. Rigoutsos I. (2015). Beyond the One-Locus-One-miRNA Paradigm: microRNA Isoforms Enable Deeper Insights into Breast Cancer Heterogeneity. Nucleic Acids Res. 43, 9158–9175. 10.1093/nar/gkv922 26400174 PMC4627084

[B37] Theocharis A. D. Skandalis S. S. Gialeli C. Karamanos N. K. (2016). Extracellular Matrix Structure. Adv. Drug Deliv. Rev. 97, 4–27. 10.1016/j.addr.2015.11.001 26562801

[B38] Tong Z. Cui Q. Wang J. Zhou Y. (2019). TransmiR v2.0: an Updated Transcription Factor-microRNA Regulation Database. Nucleic Acids Res. 47, D253–D258. 10.1093/nar/gky1023 30371815 PMC6323981

[B39] van der Kwast R. V. C. T. Woudenberg T. Quax P. H. A. Nossent A. Y. (2020). MicroRNA-411 and its 5′-IsomiR Have Distinct Targets and Functions and Are Differentially Regulated in the Vasculature under Ischemia. Mol. Ther. 28, 157–170. 10.1016/j.ymthe.2019.10.002 31636041 PMC6953895

[B40] Virtanen P. Gommers R. Oliphant T. E. Haberland M. Reddy T. Cournapeau D. (2020). SciPy 1.0: Fundamental Algorithms for Scientific Computing in Python. Nat. Methods 17, 261–272. 10.1038/s41592-019-0686-2 32015543 PMC7056644

[B41] Wang S. Yan S. Zhu S. Zhao Y. Yan J. Xiao Z. (2018). FOXF1 Induces Epithelial-Mesenchymal Transition in Colorectal Cancer Metastasis by Transcriptionally Activating SNAI1. Neoplasia 20, 996–1007. 10.1016/j.neo.2018.08.004 30189360 PMC6134153

[B42] Yang X. Chen L. Mao Y. Hu Z. He M. (20202020). Progressive and Prognostic Performance of an Extracellular Matrix-Receptor Interaction Signature in Gastric Cancer. Dis. Markers 2020, 1–23. 10.1155/2020/8816070 PMC7647771 33178362

[B43] Yang Z. Zhang C. Qi W. Cui C. Cui Y. Xuan Y. (2018). Tenascin-C as a Prognostic Determinant of Colorectal Cancer through Induction of Epithelial-To-Mesenchymal Transition and Proliferation. Exp. Mol. Pathol. 105, 216–222. 10.1016/j.yexmp.2018.08.009 30170017

[B44] Zhiyanov A. Nersisyan S. Tonevitsky A. (2021). Hairpin Sequence and Structure Is Associated with Features of isomiR Biogenesis. RNA Biol. 1, 1–9. 10.1080/15476286.2021.1952759 PMC8677047 34286662

