# Supplementary material for: ECM–Receptor Regulatory Network and Its Prognostic Role in Colorectal Cancer
Source: Front Genet. 2021 Dec 6;12:782699. doi: 10.3389/fgene.2021.782699 (PMC8685507; doi:10.3389/fgene.2021.782699)
Supplement: Supplementary file 4 [file DataSheet1.PDF]

Supplementary Material

1 Supplementary Data

JSON configuration for miRGTF-net execution:


```
{
    "interaction_databases_path": "/path/to/interactions",
    "sign_constraints": {
        "isomir_gene_interactions.tsv": "-"
    },
    "gene_expression_table_path": "/path/to/gene_expr.tsv",
    "miRNA_expression_table_path": "/path/to/isomiR_expr.tsv",
    "output_path": "/path/to/results",
    "Spearman_correlation_cutoff_percentile": 90,
    "incoming_score_threshold": 0.3,
    "interaction_score_cutoff_percentile": 10
}
```


JSON configuration for ExhauFS execution:


```
{
  "data_path": "/path/to/data.csv",
  "annotation_path": "/path/to/annotation.csv",
  "n_k_path": “/path/to/n_k.csv",
  "output_dir": "/path/to/results",

  "feature_pre_selector": "",
  "feature_selector": "cox_concordance",
  "feature_selector_kwargs": {},
  "preprocessor": "",
  "preprocessor_kwargs": {},

  "model": "CoxRegression",
  "model_kwargs": {},
  "model_CV_ranges": {},
  "model_CV_folds": 2,

  "scoring_functions": ["concordance_index", "dynamic_auc", "hazard_ratio", "logrank"],
  "main_scoring_function": "concordance_index",
  "main_scoring_threshold": 0.5,

  "n_processes": 8,
  "random_state": 17,
  "verbose": true
}
```

2 Supplementary Figures and Tables

2.1 Supplementary Figures

Supplementary Figure 1. Performance of the prognostic signatures on the training and filtration sets. A-D. hsa-miR-32-5p|0, NR1H2, SNAI1 signature. **E-H.** AGRN, DAG1, FN1, ITGA5, THBS3, TNC signature. Red point on the ROC curve corresponds to the risk score threshold, calculated as a median score on the training set.

2.2 Supplementary Tables

Supplementary Table 2. Values of *n, k* used for the exhaustive search.

Supplementary Table 2. Out-degree enrichment analysis for the ECM network.

Supplementary Table 3. In-degree enrichment analysis for the ECM network.

Supplementary Table 4. Accuracy scores of the prognostic signatures.

Supplementary Table 5. DAVID enrichment analysis of has-miR-32-5p, SNAI1 and NR1H2 target genes.

Supplementary Table 6. Summary references list on the role of the nine prognostic molecules in colorectal cancer.
