# Supplementary figures and images for: ECM–Receptor Regulatory Network and Its Prognostic Role in Colorectal Cancer
Source: Front Genet. 2021 Dec 6;12:782699. doi: 10.3389/fgene.2021.782699 (PMC8685507; doi:10.3389/fgene.2021.782699)

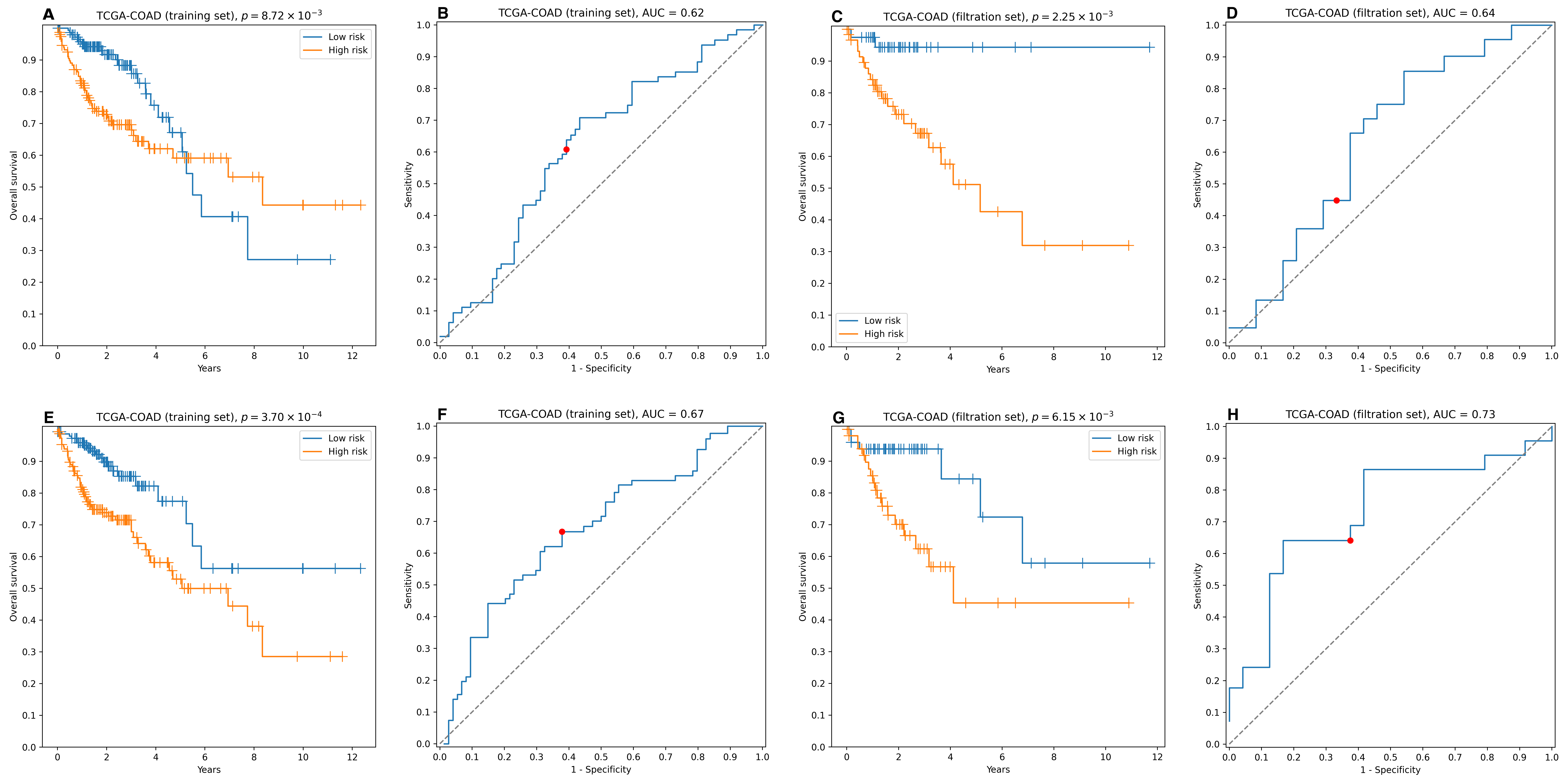

Supplement: Supplementary file 7 [file Image1.PNG]
